# Head Lice Surveillance on a Deregulated OTC-Sales Market: A Study Using Web Query Data

**DOI:** 10.1371/journal.pone.0048666

**Published:** 2012-11-07

**Authors:** Johan Lindh, Måns Magnusson, Maria Grünewald, Anette Hulth

**Affiliations:** 1 Swedish Institute for Communicable Disease Control, Solna, Sweden; 2 Department of Microbiology, Tumor and Cell Biology, Karolinska Institutet, Stockholm, Sweden; University of Ottawa, Canada

## Abstract

The head louse, *Pediculus humanus capitis*, is an obligate ectoparasite that causes infestations of humans. Studies have demonstrated a correlation between sales figures for over-the-counter (OTC) treatment products and the number of humans with head lice. The deregulation of the Swedish pharmacy market on July 1, 2009, decreased the possibility to obtain complete sale figures and thereby the possibility to obtain yearly trends of head lice infestations. In the presented study we wanted to investigate whether web queries on head lice can be used as substitute for OTC sales figures. Via Google Insights for Search and Vårdguiden medical web site, the number of queries on “huvudlöss” (head lice) and “hårlöss” (lice in hair) were obtained. The analysis showed that both the Vårdguiden series and the Google series were statistically significant (p<0.001) when added separately, but if the Google series were already included in the model, the Vårdguiden series were not statistically significant (p = 0.5689). In conclusion, web queries can detect if there is an increase or decrease of head lice infested humans in Sweden over a period of years, and be as reliable a proxy as the OTC-sales figures.

## Introduction

The head louse, *Pediculus humanus capitis*, is an obligate ectoparasite that causes infestations of humans. The parasite has been with us since before the modern man, *Homo sapiens,* diverged, and it has been proposed that both *Homo erectus* and *Homo neanderthalis* were infested with lice [1]. Human head lice live only on the human head and quickly die if they are removed [2]. Head lice infestations are mostly asymptomatic, although skin irritation and occasional secondary infection from scratching may occur [3]. Recently it has also been demonstrated that head lice *in vivo* can contain the bacteria, *Bartonella quintana* [4]. Even if no epidemiological data can be found, it is not debatable to say that head lice are the most common parasite in Europe and that they mainly infest children [5], [6], [7]. The parasites are spread between hosts via direct contact and are more common among girls [7]. In a study from Germany a clear seasonal variation in sale reports of number of pediculocides was seen during August/September and January/February [8]. This can be explained by the increases of contacts during school start, mainly between children [9]. The following decrease in sales can be explained by increased awareness, rapid diagnosis and treatment with pediculocides [8], [10]. The number of pediculocides sold should therefore follow the demand and reflect the actual occurrence of head lice in the society over time [8]. The Swedish Institute for Communicable Disease Control (SMI) follows the prevalence of head lice in society. This surveillance aids in tracking the level of treatment resistance, recommending adequate preventive measures and evaluating the effect of these recommendations.

Previously, due to cost, Over-the-Counter (OTC) sales figures for treatment products were the only practically feasible source of information for head lice prevalence in Sweden. However, with the deregulation of the Swedish pharmacy market on July 1st 2009, the number of different outlets went from one to several. Therefore, the possibility to obtain complete sales figures for the whole country disappeared.

At SMI, a surveillance system based on queries submitted to a Swedish medical web site (www.vardguiden.se) has previously been developed [11]. The Vårdguiden website is owned by the Stockholm County Council and provides various kinds of medical information in Swedish for the county’s citizens. SMI has been given access to the query logs for the Vårdguiden website. The web query-based system is used at SMI as a complement to the regular surveillance of influenza [12], [13] and of norovirus [14]. Another part of the web query-based system allows for time series to be generated for any query terms. The system contains data from June 2005.

In the presented study we wanted to investigate whether web queries on head lice can be used as a substitute for OTC sales figures, as these figures are no longer comprehensive. Furthermore, we investigated whether there is a correlation between usages of different search engines with terms on head lice, and sales of pediculocides in Sweden and whether there is any difference between the Swedish web search engine “Vårdguiden” and Google web searches with respect to this query.

**Figure 1:**
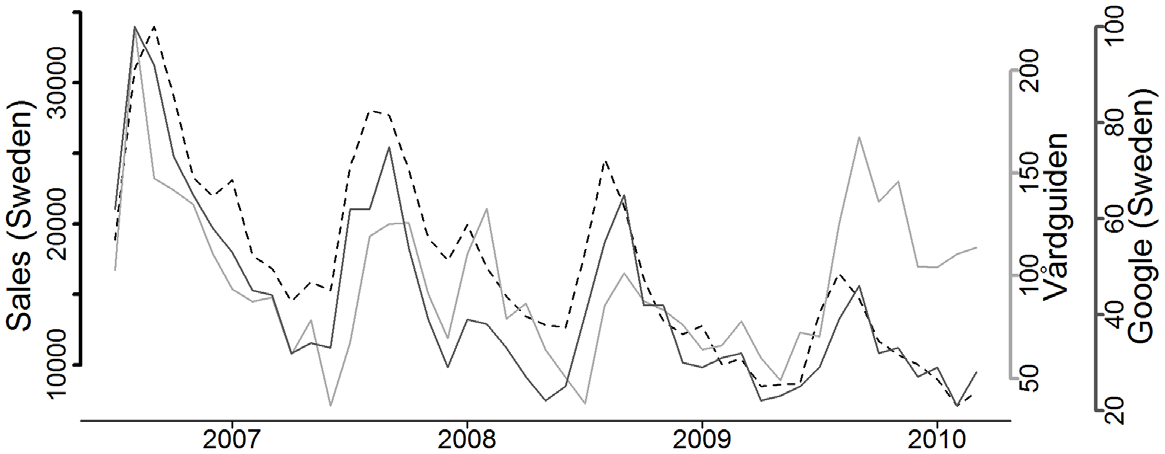
Web queries and sales (January 2006–June 2010). Dotted line – Sales (Sweden), light grey line – Vårdguiden, black line – Google (Sweden).

## Materials and Methods

### Data Sources

In the presented study, we used query data from two different kinds of websites; the medical website Vårdguiden and the general purpose search engine (Google). As the Vårdguiden logs do not contain any geographical information, the data are aggregated on a national level. Google Insights for Search is a service provided by Google where a user can obtain the relative search load on queries submitted to the Google search engine [15].

We extracted the number of queries on “huvudlöss” (head lice) and “hårlöss” (lice in hair) submitted to the Vårdguiden medical web site for the period July 2006 until March 2010 from the query logs. Through Google Insights for Search, we obtained the relative search load on Google for Sweden on the same two queries for the same time period. The time series were based on data aggregated by month. OTC sales figures per month for pediculicides for lice treatment for the same period for Sweden were obtained from Apotekens Service AB.

**Table 1:** AIC and parameter estimates of time regression models (AR(1)).

Model	Time series comp.	AIC	(Intercept)		Trend (t)		Seasonal comp. (cos(t))	Seasonal comp. (sin(t))		Web queries		Phi
**No web queries**	None	852	15988	***			–	–		–		0.89
	Trend	838	24530	***	−348	**	–	–		**–**		0.72
	Trend and season	793	24076	***	−325	***	−797	4631	***	–		0.50
**Google**	None	799	5167	**			–	–		263	***	0.76
	Trend	787	8812	***	−130	*	–	–		253	***	0.61
	Trend and season	756	10552	***	−142	*	−452	1783	*	218	***	0.63
**Vårdguiden**	None	833	9851	*			–	–		59	***	0.91
	Trend	819	18368	***	−337	***	–	–		61	***	0.70
	Trend and season	779	19284	***	−308	***	−114	3736	***	45	**	0.58

Significance codes: ‘***’: p<0.001, ‘**’: p<0.01, ‘*’: p<0.05.

**Table 2:** Correlation matrix, web queries and sales in Sweden, for lags 0, 1 and 2.

Correlation	Vårdguiden	Google	Sales	Vårdguiden,lag 1	Google,lag 1	Sales,lag 1	Vårdguiden,lag 2	Google,lag 2	Sales,lag 2
**Vårdguiden**	1								
**Google**	0.629	1							
**Sales**	0.474	0.920	1						
**Vårdguiden, lag 1**	0.600	0.367	0.286	1					
**Google, lag 1**	0.529	0.799	0.796	0.629	1				
**Sales, lag 1**	0.426	0.769	0.846	0.474	0.920	1			
**Vårdguiden, lag 2**	0.395	0.114	0.090	0.600	0.367	0.286	1		
**Google, lag 2**	0.365	0.584	0.561	0.529	0.799	0.796	0.629	1	
**Sales, lag 2**	0.313	0.576	0.617	0.426	0.769	0.846	0.474	0.920	1

### Statistical Analysis

The relationship between the sales and the web search series was analyzed with linear regression. Since the data analyzed are a time series, one of the main problems is auto correlated errors. The residuals were studied, and autocorrelation of lag one could be identified in all models. To account for this generalized least squared regression (GLS) with an AR (1)-model were used to account for auto correlated error in the statistical testing [16]. Seasonal components were used with a period of one year. Amplitude and phase were estimated from the data.

## Results and Discussion

Both the Google time series and the Vårdguiden time series were studied with regard to how well they could identify the trend and the seasonal pattern of the sales. This was done by comparing Akaike information criteria (AIC) between non nested models of sales with Google and Vårdguiden time series as covariates (Table 1). The trend was defined as a linear trend and the seasonal factor was defined as a sine/cosine function. The correlation between Google and sales was much larger than the correlation between Vårdguiden and sales (Table 2, Figure 1). The differences between the two series were tested statistically by including both series in a GLS model. The analysis showed that both Vårdguiden series and the Google series were statistically significant (p<0.001) when added separately, but if the Google series were already included in the model the Vårdguiden series was not statistically significant (p = 0.5689). The two series were studied with regarding to how well they could explain the trend and seasonal variation in the sales data. When including trend and seasonal variables in each model the model using Vårdguiden data reduced the AIC much more than the Google data series. The Vårdguiden AIC was reduced with 19 when trend was included and an additional 32 in AIC when seasonal variables were included, while the model with Google as a covariate reduced the AIC with 14 (trend) and additional 23 (seasonal variables).

In this study we investigated how well web queries can be used to follow the spread of lice in the population, by using sales as a proxy for spread of lice. Our main finding was that Google web searches are a better covariate to explain both the trend and the seasonal patterns of the sales, but that the Vårdguiden data can be used as well. A possible explanation of the outcome can be that the sales are decreasing for other reasons than a decrease in lice. Google queries and Vårdguiden queries can have different effect on the sales. For example, if one searches for lice at Google Sweden, different products are found while searching for lice on Vårdguiden rather gives the guidance of using, the relatively cheap, louse comb. This is a natural result of the two business models for the two search engines; Google is selling advertisements, while Vårdguiden has the purpose of informing the Swedish citizens. In conclusion, the queries can detect if there is an increase or decrease of head lice infested humans in Sweden over a period of years, but not the number of head lice infested people (Figure 1). The queries can also be as reliable a proxy as the OTC-sales figures for pediculicides previously were.

Google search query data have previously been shown to correlate with epidemiological data for communicable diseases, for example listeriosis [17], salmonella [18], West Nile virus [19], MRSA [20], and influenza [21], [22]. The Vårdguiden data, which originate from a medical web site, have been shown to be a valuable complement to the surveillance of communicable diseases [11], [12], [13], [14]. Web queries have also been shown to predict sales data time series for, for example, video games and cinema [23]. In this study we explored whether web queries can replace OTC sales data on head lice treatment as a proxy for head lice prevalence in society. It is our belief that there are a large number of other diseases for which web queries can complement other surveillance methods for communicable diseases. It would also be interesting to further explore the differences between query data from a general purpose search engine and a medical web site.

### Conclusions

Web queries on head lice can detect an increase or decrease of head lice infested humans in Sweden over a period of years, and be as reliable a proxy as the OTC-sales figures.
